# Increasing level of liquid pig manure reduces *Eisenia andrei* and *Enchytraeus crypticus* reproduction in subtropical soils

**DOI:** 10.1038/s41598-020-67360-4

**Published:** 2020-07-01

**Authors:** Julia Corá Segat, José Paulo Sousa, Dilmar Baretta, Osmar Klauberg-Filho

**Affiliations:** 1 Animal Science, University of Santa Catarina State, Chapecó, Brazil; 2 Centre for Functional Ecology, Department of Life Sciences, University of Coimbra, Coimbra, Portugal; 3 Center of Agriculture and Veterinary Sciences, University of Santa Catarina State, Lages, Brazil

**Keywords:** Ecosystem ecology, Biodiversity

## Abstract

Wastes generated in pig production are widely used as agricultural fertilizers. Nevertheless, such form of disposal supplies large amounts of waste in soils annually and can cause environmental pollution. The ecological risk of this practice to soil organisms has received little attention. Ecotoxicological tests are used to evaluate the toxicity of contaminants added to the soil biota. The aimed to evaluate the effect of liquid pig manure (LPM) on the reproduction of *Eisenia andrei* and *Enchytraeus crypticus* when applied in natural soils. LPM doses caused effects on earthworm reproduction in both soils, with EC_50_ of 112 and 150 m^3^ ha^−1^ in the Entisol and Nitosol, respectively. On enchytraeids, LPM had bigger effects, leading to EC_50_ of 17.7 and 45.0 m^3^ ha^−1^ in the Entisol and Nitosol, respectively. The results emphasize the importance to consider the ecological risks of LPM of conducting studies with natural soils and edaphic fauna as indicators.

## Introduction

Brazil occupies the fourth position among the main global pig meat producers^1^ and, to achieve such position, it employs a production system with high technological level and high concentration of animals in the production units. In turn, this alternative of production leads to accumulation of large volumes of pig manure per unit of area, which has been used as agricultural fertilizer, since it is an important source of N and P to agricultural soils^2^.

Soil addition of high amounts of liquid pig manure in producing regions could exceed the support capacity of the soils, which can lead to environmental damages. Prolonged utilization or large volumes of pig manure for fertilization can cause accumulation of nutrients such as P, K, Cu and Zn, especially in the 0–5 cm layer^3^; however, the norms of application do not consider the possible effects on important groups of soil organisms such as oligochaetes, which perform relevant functions in the soil, for example nutrient cycling. Therefore, the establishment of doses to be applied should also consider the possible deleterious effects on soil biota.

The magnitude of LPM application effects on survival and reproduction of edaphic organisms can be measured by ecotoxicological tests which are internationally used to evaluate toxic effects of various substances and compounds on living organisms. These tests can be used as soil quality indicators and present themselves as parameters to ecological risk analyses. Hence, organism’s responses to contaminants, obtained in ecotoxicological tests, help in the determination of safe concentrations for their use in the environment.

Earthworms (*Eisenia andrei*—Bouché) and enchytraeids (*Enchytraeus crypticus*) are good indicator organisms, because they play a key role in the provision of regulatory ecosystem services and are proven to be sensitive to the presence of certain pollutants in the soil. There are many studies in the literature evaluating the magnitude of the effects of metals, drugs, excessive nutrients and other substances on the survival, reproduction, bioaccumulation and profile of earthworm and enchytraeid communities in the soil^4–8^. However, there are few studies demonstrating the effect of using biofertilizers and organic matrices, such as liquid pig manure, in subtropical soils on these organisms^9^.

Based on the liquid pig manure composition, rich in nutrients and heavy metals (Cu and Zn), and on the current knowledge about the deleterious effects of these components on oligochaetes and about different contaminant support capacity in different soils, the hypothesis of the present study is that liquid pig manure application in subtropical soils reduces the reproduction rate of earthworms and enchytraeids. Therefore, the objective of this study was to evaluate the effect of liquid pig manure application on the reproduction of *E. andrei* earthworms and *E. crypticus* enchytraeids in two subtropical soils predominant in pig production areas in southern Brazil (Entisol and Nitosol).

## Results

All tests reached the validation criteria determined by the ISO norms for each organism in TAS. For earthworms, the average number of juveniles in the control was 569.4, with coefficient of variation (CV) of 18.5%. In the test with enchytraeids, the average number of juveniles was 437.6, with CV of 18.6%.

**Figure 1:**
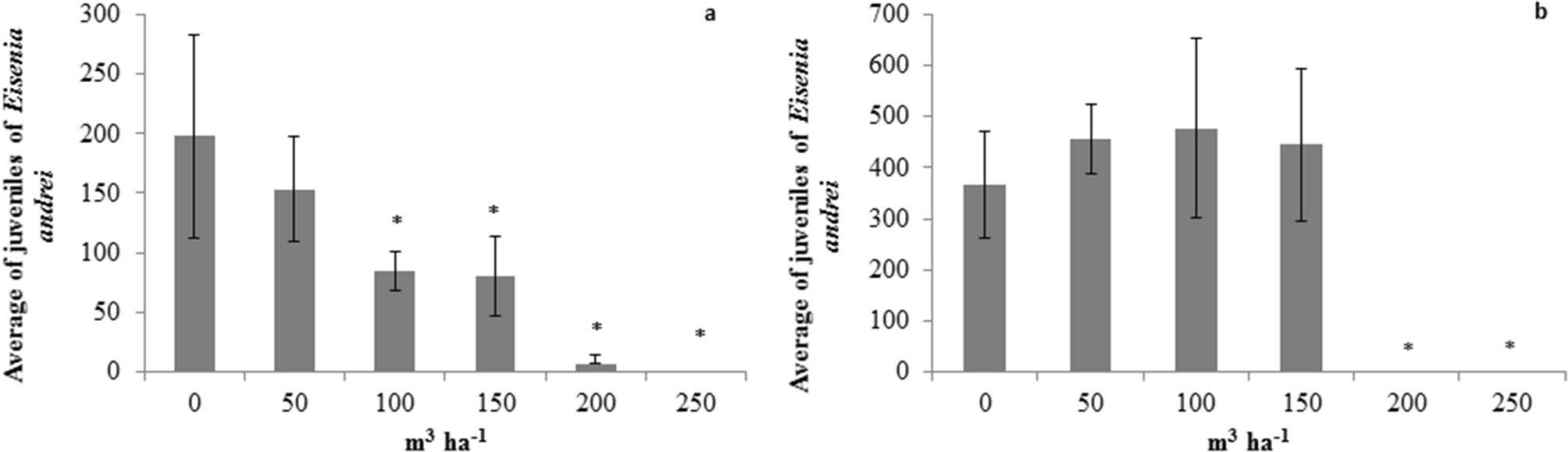
Average number of *Eisenia andrei* juveniles in the Entisol (**a**) and Nitosol (**b**) subjected to increasing doses of liquid pig manure (LPM). *Significant statistical difference (*p* < 0.05) by Dunnett’s test.

*E. andrei* reproduction decreased with the increase in LPM doses in both soils (Fig. 1a,b). In the Entisol, there were significant reductions in the number of juveniles from the LPM dose of 100 m^3^ ha^−1^ (Fig. 1a) and the observed reduction was close to 48% in the number of juveniles generated in F1. The EC_50_ calculated for this soil was 112 m^3^ ha^−1^ of LPM [95% confidence interval (CI) 76.4–147.6 m^3^ ha^−1^].

In the Nitosol, these reductions occurred from the LPM dose of 200 m^3^ ha^−1^ (Fig. 1b), at which no new individual was generated. Due to the distribution of these data, it was not possible to calculate EC_50_; however, it is possible to claim that it is higher than 150 m^3^ ha^−1^.

**Figure 2:**
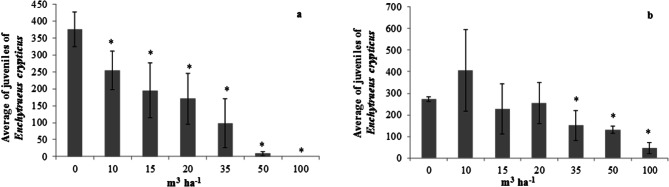
Average number of *Enchytraeus crypticus* juveniles in the Entisol (**a**) and Nitosol (**b**) subjected to increasing doses of liquid pig manure (LPM). *Significant statistical difference (*p* < 0.05) by Dunnett’s test.

Effect of LPM addition was greater on enchytraeids than on earthworms, demonstrating that these organisms are more sensitive to the organic matrix application. The results for these organisms showed even greater difference between both soils evaluated. In the Entisol (Fig. 2a), significant effect occurred already at the first dose (10 m^3^ ha^−1^), with 32% of reduction in the number of new individuals. Such effect is bigger than that found in the Nitosol (Fig. 2b), which showed effect at dose of 35 m^3^ ha^−1^, with 45% reduction in F1 individuals. As a consequence of the results, the large difference between both soils was also noted in the calculated EC_50_ values, which were 17.7 m^3^ ha^−1^ (CI 10.8–24.5) for Entisol and 45.0 m^3^ ha^−1^ (CI 30.1–59.8) for Nitosol.

## Discussion

Liquid pig manure application caused reduction in the reproduction of both studied species, *E. andrei* and *E. crypticus*. However, enchytraeids were more sensitive than earthworms in both soils, which can be noted by the calculated EC_50_ values. Evaluations performed with organic matrices are complex, due to the composition of this material, since in general, they present more than one type of contaminant and they act in a synergistic way, making it difficult to understand the real way that causes toxicity.

There are few studies evaluating the effect of organic matrices on oligochaetes. Coulibaly and Bi^10^ valuated the effect of different residues from livestock activity, observed that the lowest number of cocoons produced by *Eudrilus eugeniae* earthworms reduced by 40% with the addition of poultry litter in artificial soil. Renaud^11^ demonstrated the negative effect of pig manure on *E. fetida* reproduction which was reduced at the equivalent dose of 48 t ha^−1^, when this was applied in soil from temperate regions.

The results of the study show that earthworms were affected in doses greater than those recommended for use in the field (50 m^3^ ha^−1^), which demonstrates greater safety in the use of this waste for this group of individuals, under the conditions evaluated. In contrast, Segat^9^ observed no effects on survival, reproduction and avoidance of *E. andrei* in Latosol and Argisol contaminated with up to 100 m^3^ ha^−1^ of liquid pig manure and attributed such effect to the improvements in the conditions of the testing environment, promoted by the supply of organic matter, which can be food source. However, this situation must be carefully evaluated, since native species can be affected differently from the reference species *E. andrei* and this can cause even greater impacts on the ecosystem that is applied LPM. In a work evaluating the effect of LPM on native edaphic fauna Segat et al.^12^ pointed to a reduction of almost half in the number of oligochaetas with the application of 50 m^3^ ha^−1^, a study carried out with Nitosol in an area under crop-livestock integration system. Similar results are reported by Alves et al.^13^ where application of 100 m^3^ ha^−1^ of LPM reduced the total diversity of edaphic organisms in areas under corn cultivation and pasture.

On the other hand, enchytraeids were more sensitive to the application of LPM when compared witn earthworms. For enchytraeids in temperate soil regions, Renaud^11^ is the only study evaluating the effect of pig manure application. These authors tested the application of eight different organic wastes including composted pig manure. In this study, the calculated EC_50_ of 11.7% of composted pig manure in the soil (dry matter basis), which would represent approximately 230 t ha^−1^, is a value much higher than those found in the present study. In contrast with our results, Renaud^11^ observed higher sensitivity of earthworms (*E. fetida*), in comparison to enchytraeids (*E. crypticus*). However, Santorufo^14^, evaluating urban soils (collected in Italy) contaminated with heavy metals, demonstrated that *E. crypticus* reproduction was more affected than that of *E. andrei* in soils contaminated with 142 µg Zn and 43.8 µg Cu kg^−1^ of soil, the main metals present in the LPM of the present study.

The presence of metal has been pointed out as an important aspect of LPM composition, specially the concentrations of Zn and Cu^3,9,15^. About the effect of metals on the reproduction of earthworms and enchytraeids, Dominguez-Crespo^8^, reported that doses of 82 mg kg^−1^ of Zn and 323 mg kg^−1^ of Cu caused reduction in the number of *Eisenia fetida* cocoons as well as delay in sexual maturation. Pardo^16^ also found inhibition in the reproduction rate of *E. fetida* exposed to mine soils contaminated by different contents of Zn, Cu and Mn (1,000 mg of Zn, 0.34 mg of Cu and 246 mg of Mn per soil kg). César^17^, evaluating the transport of metals (which could suggest sublethal effects) from contaminated soils of mining areas to *E. andrei*, observed that Zn and subsequently Cu were the metals with highest capacity of transport to the organisms.

In the present study, the estimated contents, at the lowest dose of LPM with significant effect in enchytraeids (10 and 35 m^3^ ha^−1^), corresponded to the concentrations of Cu and Zn of 1.3 and 11.4 mg kg^−1^ of Entisol soil; and 4.8 mg of Cu and 39.8 mg of Zn per kg^−1^ for Nitosol soil, which are lower than those found by Lock and Janssen^18^, evaluating the reproduction of enchytraeids exposed to contamination of 305 mg kg^−1^ of Cu and 97 mg kg^−1^ of Zn. Reproduction rate reduction has also been observed in soils contaminated with 267 and 345 mg kg^−1^ of Zn^18,19^, but Onuoha and Worgu^7^ state that the combination of metals may have an additive effect on toxicity when compared to pure metals.

Effects of metals on earthworms and enchytraeids are not limited to their reproduction. Studies have shown that there might be effects on survival rate, such as Onuoha and Worgu^7^, who found mortality increased when of *E. andrei* were exposed to 1,000 mg of Zn. Natal-da-Luz^6^ found LC_50_ (Lethal concentration) for *E. andrei* of 79.3 mg of Cu and 397 mg of Zn kg^−1^ of soil. Amorim^20^, evaluating the effect of Zn and Cu on *Enchytraeus albidus* avoidance, found significant effect at doses of 92 and 133 mg kg^−1^ of soil. Effect of metals on natural enchytraeid communities was reported by Tosza^5^. These authors evaluated Zn and Cu contaminated areas and found lower diversity and density of enchytraeids when contamination was from 612 to 2,629 mg of Zn and from 56 to 216 of Cu.

Of the other the compounds that are present in the LPM, and that can cause negative effects on earthworms and enchytraeid, we can highlight nitrogenous compounds such as ammonia. Ammonia has already been reported in the literature as causing a reduction in the survival and reproduction of edaphic organisms as is the case of springtails^21^ and mites^11^. In work by Renaud^11^, the authors state that ammonia is toxic to earthworms and enchytraeid when applying organic residues to the soil. For the present study, the concentrations of N–NH_4_ from the application of LPM were not evaluated, but the total N load that was applied in doses that showed a significant effect is high.

The application of organic compounds in the soil can increase the concentration of organic acids in the system, especially those of low molecular weight, due to the decomposition process that this material undergoes^22,23^. Although the effects of adding organic acids on the development of earthworms and enchytraeids have not been found, the effects caused by these components can be indirect. The presence of organic acids can cause the dissolution of the mineral structure of the soil causing the contaminants to become more bioavailable since these will have fewer places to adsorb to the soil^24^.

In addition to the effect of the presence of metals, ammonia and organic acids in the LPM composition, the soil in which these organic matrices will be applied has a great influence on the dynamics of the effects of toxicity, since the characteristics of this soil can act as facilitators or not of the retention of the contaminants leaving them less available for effects in edaphic organisms. Based on the results, it is possible to identify differences among both studied soils concerning waste retention, which could be causing higher toxicity in the Entisol, for the two organisms tested. These results can be directly related to the characteristics of the soils. Among the soil characteristics that can influence the toxicity effects of LPM, the main ones are clay and organic matter content. It can be observed in Entisol that has lower values of clay and organic matter than Nitosol.

The differences found for the same organism, between both soils evaluated, Entisol and Nitosol, may be an effect of the organism’s capacity to develop under certain characteristics of the soil, as reported by Amorim^25^, who demonstrate that the exposure of enchytraeids to chemical compounds in different soils causes different levels of toxicity. However, this effect was not observed in the present study, because the organisms reproduced at satisfactory rate in the soils that did not receive waste.

Different effects between the evaluated soils occurred because one of them had lower clay content, which may result in lower adsorption of certain metal cations in the clay minerals, making them more available, thus affecting the reproduction of earthworms and enchytraeids. These effects have been demonstrated in studies such as Sivakumar and Subbhuraam^26^ with Cr(III) and Cr(VI)^27^, and Maboeta^28^ with Cu and Reinecke^29^ with Zn. Gräber^30^ claim that Cu and Zn mobility and availability, for instance, are influenced by soil characteristics, including clay content.

In the specific case of pig manure addition to the soil, it is already known that soils with lower clay content have higher availability of metals, in comparison to soils with higher clay contents^3^. Mattias^31^ evaluated the availability and accumulation of Cu, Zn and Mn in Latosol, Luvisol and Entisol of Santa Catarina, with successive pig manure applications, and found higher risk of environmental contamination in Entisol, attributing it the lower initial content of organic matter. The same result can be observed in the data of the present study, in which Entisol showed toxic effects at lower doses of LPM, in comparison with Nitosol, which is a response of the combination of the difference of the contents of organic matter and the small difference of the % clay between soils. In study about LPM effect on *E. andrei*, Segat^9^ found reduction in reproduction at LPM doses of 30 m^3^ ha^−1^ in Neosol.

The results obtained in the present study demonstrate that liquid pig manure causes reduction in the reproduction rate of *E. andrei* earthworms and *E. crypticus* enchytraeids, and this effect occurs differently in soils of different classes. When we think of a condition at the field level, it is known that the use of LPM or other organic residue alters the composition of the edaphic fauna as a whole, promoting the local extinction of some species or groups and favoring the appearance of others, and these situations can be extrapolated to earthworms and enchytraeids^12,19^. In addition, LPM is currently used as an agricultural fertilizer and due to this practice, sequential applications are carried out over years, which can cause the effects to be cumulative over the time of use of this residue in the soil, mainly due to the accumulation and increased availability of toxic compounds^12^. However, the present study evaluated the effects caused by a single application to the soil and over a period that comprised part of the life cycle of the organisms in question.

## Methods

The organisms were obtained from pure cultures of the Soil and Sustainability Laboratory of the Santa Catarina State University—Western Higher Education Center (UDESC/CEO), according to the guidelines established by the ISO 11268-2^32^ for *Eisenia andrei* earthworms and ISO 16387^33^ for *Enchytraeus crypticus* enchytraeids. The organisms were weekly fed a cooked mixture of rolled oats and distilled water at proportion of 2:1 (v/v) and maintained in environment with controlled temperature of 22 °C ± 2 and photoperiod of 12 h. The tests were conducted using organisms less than 6 months old and with apparent clitellum.

Soil samples used in the ecotoxicological tests were collected in Crop-Livestock Integration areas without history of pig manure utilization in the last 10 years. Two different natural soils were used: dystroferric Red Nitosol (Nitosol), from the municipality of Concórdia-SC and eutroferric Haplic Entisol (Entisol), from Chapecó-SC. Both were collected in the 0–0.20 m layer, dried in an oven at 65 °C and sieved through a 2-mm mesh. Their physical–chemical parameters are presented in Table 1.

**Table 1:** Physical–chemical characteristics of the eutroferric Haplic Entisol (Entisol), dystroferric Red Nitosol (Nitosol) and Tropical Artificial Soil (TAS).

Soil	Clay (%)	CEC^a^	SB^b^	pH (H_2_0)	OM (g dm^-3^)	P	K	Ca	Mg	H + Al	Cu	Zn	Fe
		(cmolc dm^-3^)				(mg kg^−1^)		(cmolc dm^-3^)			(mg kg^−1^)		
Entisol*	31	11.5	24.9	5.6	1.3	1.2	36	17	10	87	127	78.5	78.2
Nitosol*	33	18.9	13.9	5.5	4.7	6.8	353	8.2	5	5	15.7	20	201.1
TAS	20	70.5	48.5	6.2	49	39	25.5	14	9	22	0.2	0.7	3

^a^Cation Exchange Capacity.

^b^Sum of Bases.

*According to the Brazilian Soil Classification System (EMBRAPA, 2006).

For control purposes, a Tropical Artificial Soil (TAS) was also used, composed of a mixture of 70% industrial sand (fine), 20% kaolinitic clay and 10% coconut shell (dried and sieved)^34^. The pH values in the TAS were corrected to 6.0 ± 0.5 by adding CaCO_3_ and the moisture was corrected to 60% of the water retention capacity of each soil and each pig manure concentration, at the beginning of the tests.

The liquid pig manure (LPM) used in the tests was directly collected in a finishing pig production unit of the EMBRAPA Swine and Poultry—Concórdia, SC. LPM was subjected to stabilization for 120 days, as recommended by the Commission of Chemistry and Soil Fertility^2^, and its physical–chemical characteristics are in Table 2.

**Table 2:** Physical–chemical characteristics of the fresh liquid pig manure collected in the finishing stage.

pH (CaCl_2_)	Moisture (%)	Total N	P_2_O_5_	K_2_O	Cu	Zn	Fe	Mn
		(kg m^-3^)			(g m^-3^)			
6.5	82	6.8	0.5	3.6	27.7	227.9	309.2	77.4

The LPM doses used in the ecotoxicological tests in Nitosol and Entisol were calculated based on the recommendations of the Normative Instruction No. 11^35^ for the utilization of animal manure as agricultural fertilizer, which recommends the application of 50 m^3^ of pig manure ha^−1^ year^−1^. LPM doses were equal to 0, 50, 100, 150, 200, 250 and 300 m^3^ ha^−1^ in the tests with earthworms and to 0, 10, 15, 20, 35, 50 and 100 m^3^ ha^−1^ in the tests with enchytraeids, due to the higher sensitivity of the latter. The respective doses were incorporated into the soil and later placed in the test containers.

The test of chronic effect on earthworms followed the recommendations of ISO 11268-2^32^. Each experimental unit, was made up of a plastic container with a capacity of 1 L, received 10 clitellated individuals, which remained for 28 days. After this period, they were removed, leaving only soil, cocoons and juveniles in the containers. After 56 days from the beginning of the test, the number of juveniles was counted in each experimental unit. To maintain the test, horse manure (dried, sieved and defaunated) was weekly supplied (5 g per container) and moisture was corrected in the containers based on the difference of weight, in relation to the beginning of the test.

Enchytraeid reproduction tests were set according to ISO 16387^33^. Ten clitellated adult individuals were placed in a plastic container (0, 150 L) containing the soil and LPM and maintained for 28 days. After this period, the containers received alcohol and Rose Bengal dye to color the enchytraeids for the count. During the experimental period, the organisms were fed with rolled oats and the moisture was corrected every week.

All tests were conducted with five replicates per treatment. Reproduction data of the organisms were initially analyzed for normality and homogeneity of variances using the Kolmogorov–Smirnov and Levene’s tests, respectively. Subsequently, the data were subjected to one-way analysis of variance (ANOVA) and Dunnett’s test to compare the means using the Software SAS 9.2. Logistic regression analyses were performed in the program STATISTICA^®^7.0, according to Environment Canada^36^, to determine EC_50_ values (effective concentration—with significant effect on 50% of the individuals) in the chronic toxicity tests.

## Data Availability

All data generated or analyzed during this study are included in this published article (and its Supplementary Information files).
